# 

**Published:** 1908-07-15

**Authors:** 


### ANNOUNCEMENTS.

THE NATIONAL DENTAL ASSOCIATION.—This and other societies which meet at the same time will meet in Boston the week of July 26th to 31st. Great preparations are being made by the New England dentists for a grand meeting and all who can attend should be greatly pleased and instructed. The following is a partial program:

Section 1 presents the following papers: Dr. H. A. Pullen, of Buffalo, N. Y., "Orthodontia." Dr. Geo. H. Wilson, Cleveland, O., "Some Phases in the Construction of Complete Vulcanite Dentures." (Exhibiting 30 to 40 slides, showing appliances and points in construction.) Dr. Charles Channing Allen, Kansas City, Mo., "Metallurgy."

Section 2 presents the following papers: Dr. Burton Lee Thorpe, St. Louis, Mo., "The Contributions of New England Dentists." Dr. Geo. W. Weld, New York City, "Characteristics of Calcified Tissues in Two Complete Sets of Human Teeth, Free from Caries." Dr. Joseph Head, Philadelphia, Pa., "The Protecting Action of Saliva from Decalcification of Enamel by Acids."

Section 3 presents the following papers: Dr. Clyde Davis, Lincoln, Nebraska, "A Method of Treatment of Purulent Eupyema of the Maxillary Sinus." Dr. Eugene S. Talbot, Chicago, Ill., "Acidosis and Indicanuria in Diseases of the Mouth, Jaws and Teeth." Dr. Thomas B. Hartzell, Minneapolis, Minn., (subject later). Dr. Arthur H. Merritt, New York City, (subject later). Nearly 150 clinics are scheduled to date and more are expected.

*Railway Rates.* New England Association, which includes the territory of New England, grants a rate of a fare and one-third on certificate plan. Trunk Line Association, which includes the territory of Buffalo, Niagara Falls, N. Y., Erie and Pittsburg, Pa., Bellaire, O., Wheeling, Parkersburg and Huntington, W. Va., and points east thereof, except New England, a rate of one and three-fifths, also on certificate plan. All other territory grants summer tourists rates in existence and effective to Boston during the summer. Those using the Trunk Line rates will deposit their certificate, together with 25 cents, with the secretary, Dr. C. S. Butler, immediately on arriving at the meeting.

—✦—

INVITATION TO THE CANADIAN DENTAL ASSOCIATION.—The Canadian Dental Association meets in Ottawa August 4, 5, 6, 1908, and extends an invitation to the members of the National Dental Association of the United States to attend its meeting. Arrangements have been made with the Canadian Pacific Railway Company that all tickets from the west over the Canadian Route, to Boston, will be honored via Ottawa on the return trip.

This will give those attending the National Dental Association an opportunity to visit the St. Lawrence Route, City of Montreal and the Capital City of Canada and in this position be close to what has been termed the play ground of America. No better hunting, fishing and camping can be found than what is within the reach of the city of Ottawa. Besides the Canadian Dental Association would feel it a great honor to have with them any of their confreres of the United States who choose to accept this invitation.

—✦—

CINCINNATI COLLEGE OF DENTAL SURGERY.—*Graduates.*—E. R. Cain, J. C. F. Craig, F. E. Dellinger, J. Eaton, W. F. Grossmann, F. I. Hage, E. W. Hill, F. P. Lang, C. E. Machle, M. D., E. McK. Miller, N. E. Shai, J. N. Smith, T. J. Welsh.

—✦—

OHIO COLLEGE OF DENTAL SURGERY.—*Graduates.*—C. Baer, R. E. Barton, A. Beagle, E. M. Beddow, G. L. Brunk, E. F. Cleland, E. D. East, C. G. Elder, P. A. Falbush, H. E. Germann, M. H. Glossinger, A. Hall, H. W. Hamilton, C. M. Hildebrand, J. A. Keller, Jr., C. F. Kennedy, B. Kinsley, C. J. Lindemann, W. McCaughrin, E. McCurdy, H. A. McLaughlin, J. E. McComb, W. F. Marlatt, Ph. G., F. R. Martin, M. M. Maupin, J. C. Maybin, W. P. Monohan, E. W. Neufarth, J. W. Purdy, F. C. Robinson, H. M. Schweinsberger, R. R. Shelt, E. E. Spencer, D. D. Stoner, W. C. Thompson, H. M. Thornton, J. M. Turner, C. S. Warren, D. Walton, R. C. Wells.

---

Detroit College of Medicine, Dental Department. —Becker, Arthur; Bernstein, Peter; Carleton, Roy H.; Cook, Mahlon L.; Crostick, George A.; Czubachowski, Joseph M.; Danielson, Walter F.; Finlayson, John M.; Forman, Chester L.; Gracey, Clayton H.; Greenwood, William E.; Halbstein, Louis M.; Harsen, Clark H.; Hill, Gordon W.; Housen, Wm. F.; Joy, William E.; Kearns, Wilfred W.; Kent, Sherman E.; Lawson, Hilmer L.; Lipski, Stanislaus; Ludt, Carl E.; McKenna, Archibald C.; McNeill, George V.; Mailloux, Donat D.; Martyn, Donald; Moran, Granville C.; Tucker, Charles H.; Wettlaufer, Edward J.

---

University of Michigan, College of Dental Surgery.—*Doctor of Dental Surgery.*—G. G. Andree, B. S., Columbian University, G. H. Attarian, W. R. Barney, J. D. Bentley H. E. Brady, E. S. Braithwaite, H. W. Brouse, F. W. Bryant, W. J, Burley, E. F. Burns, A. E. Camp, G. O. Clark, G. R. Clark, J. A. Connery, Jr., A. H. de Goenaga, E. A. de Goenaga G. W. Dunham, B. R. East, J. G. Erwin, R. Evans, F. J. Farthing, D. Faucher, H. J. Fox, H. A. Goodwin, E. L. Hering, J. J. Jacobs, E. T. Keating, H. D. Keenan, E. G. Keith, J. E. Kilgore, H. E. Kreager, F. E. MacMullen, H. J. Maher, M. M. Main, B. H. Masselink, C. N. Merritt, P. D. Miller, J. G. Morningstar, W. B. O'Neill, H. F. Parks, W. J. Plunkett, C. W. Pratt, J. C. Ranger, J. E. L. Richmond, B. A. Roelofs. C. A. Rysdorp, H. D. Schweinhagen, R. C. Simmons, P. W. Simpkins, R. W. Thomas, C. L. Thompson, L. Tregea, W. J. A. Wagner, H. E. Wright. *Doctor of Dental Science.*—Russell Welford Bunting, D. D. S.

